# Soliciting organ donations by medical personnel and organ donation coordinators: A factor analysis

**DOI:** 10.1371/journal.pone.0250249

**Published:** 2021-04-23

**Authors:** Yao-Mei Chuang, Shan-Shan Yeh, Chi-Fen Tseng, Chie-Chien Tseng

**Affiliations:** 1 Department of Health Promotion and Health Education, National Taiwan Normal University, Taipei, Taiwan; 2 Department of Nursing, Tzu Chi University of Science and Technology, Hualien, Taiwan; 3 Organ Coordinator of the Third Military General Hospital, Taipei, Taiwan; Imperial College Healthcare NHS Trust, UNITED KINGDOM

## Abstract

The literature on organ donation in Taiwan lacks a discussion of the roles of medical staff, organ donors, and transplant coordinators in organ donation. The biggest plight of organ donation is lack of the organ donations. When we probed the possibilities of not finish the organ donation procedure, such as religions, traditions and cultural belief, disease cognitions, and the failure of persuasion or the loss of organ donors. There are lots of research literature shown that the attitude of medical personnel would influence the willingness of organ donation or persuasion. This study considered such personnel and their participation in organ donation, specifically analyzing factors influencing their effectiveness. Snowball sampling was adopted to recruit medical staff, organ donors, and transplant coordinators for an online survey. The results revealed that some participants were unclear as to how to initiate the organ donation process and what practical operations are involved. Even with the necessary qualifications, some participants remained passive when soliciting organ donations in clinical practice. Organ donation coordinators with experience in caring for organ donors who attended organ donation courses performed well in soliciting organ donations. The researchers recommend that training courses on clinical planning and organ donation are incorporated into intensive care training and that they serve as the basis for counsellors soliciting organ donations to increase nurses’ willingness to solicit organ donations.

## Introduction

Much of the relevant literature in Taiwan and abroad argues that the attitude of medical staff toward potential organ donors affects actual donation or solicitation. If medical staff solicit donations with confidence and awareness such that patients’ families do not reject the idea of organ donations, 84% of family members may agree to organ donations when medical staff proactively ask for it. Conversely, family members may unanimously refuse when asked without confidence or warning. This proves that the attitudes and thoughts of medical staff affect the decisions of patients’ families, highlighting how medical staff members’ past experiences, educational background, personal perception, willingness, and attitude toward organ donation and transplants may affect the discovery of potential donors [1,2].

Collating the literature on organ donation in Taiwan revealed a lack of discussion of the direct effects of medical staff, organ donors, and transplant coordinators on organ donations. Most papers have only focused on nursing staff [1]. Furthermore, studies have reported that although domestic nursing staff display positive attitudes toward organ donation, a majority express an inability to decide if they are willing to donate their own organs or exhibit little willingness [2]. Further discussion rarely occurs. Moreover, organ donation and transplant coordinators are often first-line personnel in the entire organ donation and transplant procedure. Other than coordinating communications across medical teams, key tasks include assisting potential organ donors to become actual donors, soliciting those with viable organs and tissue for donation, and participating and monitoring the organ retrieval process and the safe and successful transplantation of healthy organs and tissue [1].

This study considered front line personnel in organ donations—medical staff and organ donation and transplant coordinators—and their execution of organ donations to evaluate factors influencing their effectiveness.

## Literature review

Organ transplants have a long history in Taiwan. In 1968, Professor Chun-jean Lee of National Taiwan University Hospital successfully completed the first kidney transplant in Asia and opened the door for organ transplants in Taiwan. Following the enactment of the 1987 Human Organ Transplant Act as well as procedures for determining brain death, Taiwan became the first country in Asia to establish regulations governing organ transplants (and its technology), which resulted in subsequent successes in liver, heart, and lung transplants. In contrast to Japan, which did not legislate for brain death until 1999, Taiwan is a pioneer of organ transplantation in Asia [2].

As organ transplant technology matured and developed, organ transplants became the last hope of many patients facing organ failure. With the invention of new immune suppressants and anti-rejection drugs in particular, organs such as the heart, liver, and kidneys have achieved 70% and even up to 95% 3-year survival rates. According to the Taiwan Organ Registry and Sharing Center’s statistics, over 8,000 patients are waiting for a successful organ match at 2018. Only approximately 200 organ donors appear each year. This shortage causes a substantial bottleneck in organ donations.

Despite the high survival rate of organ transplant recipients and the increasing number of people with organ donor cards in Taiwan, organ donation rates in Taiwan remain low compared with European and American countries. Spain has the most comprehensive organ donation measures in the world. It had an organ donation rate of 17.8 per million people in 1990 and 35.1 per million in 2013 [3]. This rate is five times that in Taiwan. Organ donation levels are low in Taiwan primarily because of religious beliefs, traditional customs, fear of disease, failure to recruit organ donors, and loss of potential donors [4,5]. In addition to patients’ and family members’ self-worth affecting their decisions, the decisions of such patients can be influenced by attending clinical medical staff. Many studies have reported that clinical medical staff are often the first to discover potential organ donors but seldom proactively inform organ donation and transplantation teams or inquire with family members regarding organ donations [5,6]. Article 10–1, Paragraph 4 of Taiwan’s Human Organ Transplant Act states the following: “To promote the ethos of organ donating, hospitals shall take initiatives to establish a donation soliciting mechanism to ask the family members of potential donors of suitable organs of their desire for organ donation, and hence expand the sources of organ donation.” Therefore, soliciting organ donations is a legal expectation of medical staff.

At present, in Taiwan, hospital are responsible for organ donation and its attendant procedures. Organ donation generally refers to the donation of organs or tissues following brain death or the end of life. In Taiwan, members of the organ transplant office of each hospital include transplant coordinators, social workers, and transplant nurse practitioners. Upon discovering a potential donor during clinical care, frontline medical staff immediately notify the organ transplant office to initiate the organ donation procedure. First, a transplant nurse practitioner of the office assesses whether the individual is suitable for organ donation. Subsequently, a social worker communicates with the individual’s family to understand their opinions. For the individual to donate his or her organ, the consent of his/her family members must be obtained and an organ donation consent form must be signed before the organ donation application can be processed in the subsequent medical treatment. According to Article 4 of the Human Organ Transplant Act, “when performing a transplant operation by removing an organ from a corpse, the organ donor shall be certified dead by his/her attending physician before the operation can be performed.” Brain death must be determined in accordance with the procedures stipulated by the Ministry of Health and Welfare. In addition, According to Article 12 of Human Organ Transplant Act, “any organ for transplantation shall be provided or acquired free of charge.” The stipulated organ donation procedures are as follows. First, frontline nursing staff find potential organ donors through an assessment by a doctor or by a recommendation by family members. Second, and subsequently, frontline nursing staff notify members of the organ donation office in the hospital. Third, nursing staff members explain the organ donation procedures to the family member(s) of a donor and confirm their willingness to accept the donation. Fourth, the nursing staff members guide the donor’s (patient’s) family member(s) in signing a consent form and relevant documents. Fifth, the organ donation office initiates the requisite tests, takes care of the donor, and maintains the vital signs of the patient or donor. Sixth, a determination of brain death of the donor must undergo confirm twice. Seventh, and finally, organ donation and transplant surgeries are then performed.

## Materials and methods

### Research design

With the consent of the Organ Donation Association, snowball sampling was adopted to sample medical staff and organ donation and transplant coordinators.

### Participants and eligibility criteria

The target group consisted of certified organ donation and transplant coordinators who were asked to invite a doctor (of any level—intern, resident, or attending—and discipline aged 20 years and older) and nurse (over 3 months of work experience and familiar with clinical nursing services) to complete an online survey. The online survey was distributed with assistance from the Organ Donation Association. The survey explained the research purpose 0and content to the participants. A total of 192 valid responses were collected (response rate: 73.8%). The Research Ethics Committee of the National Taiwan University approved this study (IRB:201504ES002).

### Research tools

The self-developed questionnaire was constructed with reference to the expert questionnaires used by Huang et al. [6], Shi et al. [7], Zhang et al. [8], and Cory et al. [9] and was divided into four sections. The design was based on questionnaire design principles that test hypotheses in terms easily comprehensible by interviewees to gain insight into their traits. Section 1 concerns demographic variables, and the organ donations attitude scale in Section 2 contains 20 items regarding participants’ thoughts, beliefs, and behavioral tendencies toward organ donations, including their thoughts and views on organ donations and care. Section 3 comprises 14 yes/no questions on participants’ experiences with organ donation or the organ donation knowledge scale, which surveys organ donation and transplant coordinators’ level of understanding of the definition, determination of brain death as well as related legal requirements. The organ donation efficacy scale of Section 4 focuses on participants’ successful and failed experiences in soliciting organ donations.

### Validity and reliability of research tools

#### Expert validity review

Five experts on organ donation were invited to test the validity of the first draft of the self-developed organ donation survey—an organ donation–soliciting physician, transplant surgical nurse, social worker, transplant coordinator, and family member of a patient who successfully received an organ donation. The content validity index of the survey reached 0.818.

#### Trust level analysis

After formal samples from the parent group were excluded, 35 participants were chosen for a pretest. The Cronbach’s α of the survey’s internal consistency was 0.856 for the organ donation attitude scale and 0.704 for the organ donation knowledge scale, which indicated that the survey was valid and could be adopted.

### Statistical analysis

The coding, archival, and statistical analyses of the survey responses were processed in Excel and SPSS by using descriptive (frequency distribution and percentages) and inferential statistics. Descriptive statistics of the following variables were calculated: basic characteristics (gender, age, education level, marital status, religious belief, and explicit consent given to be an organ donor), job attributes (number of years working, type of occupation, department working at, and hospital type; indicated by frequency and percentage), scores on the organ donation attitude scale, scores on the organ donation knowledge scale, and variables for performance in promoting organ donation. Regarding inferential statistics, an independent samples *t* test, one way analysis of variance, Pearson’s product–moment correlation, and multiple regression were used to analyze the relationships between basic attributes, organ donation attitude, organ donation knowledge, and performance in soliciting organ donations.

## Results

### Demographic distribution

The demographic information of the participating medical staff and organ donation and transplant coordinators were analyzed using descriptive statistics as both numbers and percentages. The results revealed that the majority of participants were female and worked in surgical disciplines, such as organ donation and transplant coordinators, or were employed in medical centers. The average age range was 30–39 years, and the participants were mostly college or technical school graduates. Most participants were married and had no religious beliefs. Most participants had 5 or more years of clinical work experience, and most had organ donor cards and attended courses on organ donation.

### Responses on the organ donation attitude scale

The highest scoring item was “I think that organ donation is meaningful” and had an average score of 4.53 (SD = 0.63). The second highest scoring item was “Organ donation continues the organ donor’s life and gives the recipient a second chance at life” and had an average score of 4.43(SD = 0.64). The third highest scoring item was “I think that coordinating the solicitation of organ donations is a meaningful task” and had an average score of 4.40 (SD = 0.67).

This section comprised 14 yes–no questions (1 point was awarded for each correct answer for a maximum score of 14) on workplace organ donation procedures and regulations for determining brain death. The three highest scoring questions were “After the first test to determine brain death, at least 4 hours must elapse before the second test is conducted in accordance with prescribed procedures”, “According to Taiwan’s standards for determining brain death, brain death is defined as brain stem death,” and “Central health and welfare agencies shall subsidize the funeral costs of organ donors as prescribed by those agencies.” These score result suggested a certain degree of understanding of the guidelines that determine brain death.

The survey revealed that 113(58.9%) participants in organ donation solicitation, and 79 (41.1%) did not. Among participants who never solicitated organ donations, the more common reasons were “This is not the business of my current department,” “I think organ donation solicitation from the organ donation and transplant team is more appropriate,” and “I have not encountered appropriate patients to solicit donations.” These results revealed that some participants were passive in advocating organ donations.

Among reasons for successful organ donations arising from active solicitation by study participants and when participants felt that patients’ families proposed the donations, the first and third most common reasons for both types of donation were “Organ donations can help other people” and “The organ donor wished to donate their organs or had signed an organ donor card.” The second most common reason was “Organ donation is a means of continuing life” for the successful solicitation of organ donations by participants and “Organ donations are acts of kindness that will be rewarded” for unsolicited donations by family members. These results revealed that most reasons for successful donations were derived from altruism.

The survey indicated reasons for failed organ donations. The top three reasons participants gave for families’ refusals were “The family members desired to keep the patient’s body intact or were unwilling to have the patient suffer from operations again,” “The family did not want to donate the patient’s organs,” and “Resuscitation was pursued to the full extent.” Thus, these reasons were consistent with those of the literature [5,10–12].

### Relationship between participant demographics and the organ donation attitude scale

Among the 13 variables for all demographic traits in Table 1, participants’ attitude toward organ donation exhibited significant differences in terms of “whether they are registered organ donors” and “whether they attended organ donation courses” (F = 15.353, *p*<0.01; *T* = 2.675, *p* = 0.008). Comparisons after a Bonferroni post hoc test revealed that unregistered and registered cardholders had higher average attitude scores than did non registrants. The average score differences were 5.18 (*p* = 0.004) and 7.23 (*p*< 0.001), respectively. The remaining variables on basic attributes were not significantly correlated with the organ donation attitude scale; these variables were sex (*F* = 0.971, *p* = 0.333), age (F = 0.977, p = 0.378), education level (F = 0.971, p = 0.381), Religious belief (F = -0.442, p = 0.659), Marital status (F = 1.391, p = 0.251), Department of work (F = 041, p = 0.872), Working years (F = 1.155, p = 0.328), Job title (F = -0.177, p = 0.859), Type of hospital (F = 1.444, p = 0.231), Take care of organs donor experience (F = -1.166, p = 0.245), Experience in caring for organ recipients(F = -1.109, p = 0.269).The complete form is as S1 Table in the attached information.

**Table 1:** Factors related to participants’ basic attributes and attitudes toward organ donation.

Basic attributes		Number	Average	Standard deviation	T value/F value	P value	Post hoc comparison test
**Whether they are registered organ donors**	①no	95	74.9	8.9	15.353	<0.001	①v.s②(p = 0.004)
	②Signed card but no note	37	80.0	7.5			①v.s.③(p<0.001)
	③Have a card and have a note on the health card	60	82.1	7.7			
**Whether they attended organ donation courses**	participated	171	78.7	8.7	2.675	0.008	
	Never participated	21	73.3	8.5			

### Relationship between participant demographics and the organ donation knowledge scale

Table 2 presents a significant difference in participants’ knowledge of organ donations because by discipline, job title (*F* = 7.97, *p*<0.001), type of hospital of employment (*F* = 7.81, *p*<0.001), experience in caring for organ donors (*T* = −4.23, *p*<0.001), and attendance at organ donation courses (*T* = 2.89, p = 0.01). Comparisons after a Bonferroni post hoc test indicated that participants working in obstetrics and gynecology had less knowledge of organ donations than did those from other disciplines. Those at primary care exhibited less knowledge of organ donations than did those from other types of hospitals. The remaining variables on basic attributes were not significantly correlated with the organ donation attitude scale; these variables were Sex (F = -0.31, p = 0.94), Age (F = 0.76, p = 0.47), Education level (F = 2.82, p = 0.06), Religious belief (F = -0.27, p = 0.79), Marital status (F = 0.64, p = 0.53), Working years (F = 0.77, p = 0.51). The complete form is as S2 Table in the attached information

**Table 2:** Factors related to participants’ basic attributes and knowledge of organ donation behavior (*N* = 192).

Basic attributes		Number	Average	Standard deviation	T value/F value	P value	Post hoc comparison test
**Department of work**	①Emergency	7	8.6	1.6	7.97	<0.001	④v.s. ①(p = 0.019)
	②Surgery	62	9.5	1.1			④v.s. ②(p<0.001)
	③Internal medicine	42	9.0	1.0			④v.s. ③(p<0.001)
	④Obstetrics and gynecology	4	6.3	0.5			
	⑤Pediatrics	4	8.3	1.9			④v.s. ⑥(p<0.001)
	⑥Intensive care unit	43	9.4	1.3			④v.s. ⑦(p<0.001)
	⑦Other: social worker	30	9.7	0.7			
**Job title**	Medical staff (physician, nurse)	99	8.7	1.4	−4.82	<0.001	
	Organ donation coordinator	93	9.7	0.8			
**Type of hospital**	①Medical center	103	9.4	1.0	7.81	<0.001	④v.s. ①(p<0.001)
	②Regional hospital	65	9.3	1.3			④v.s. ②(p<0.001)
	③District hospital	17	9.0	1.3			④v.s. ③(p = 0.007)
	④Primary care	7	7.3	1.6			
**Take care of organs donor experience**	No	99	8.9	1.3	−4.23	<0.001	
	Have	93	9.6	1.0			
**Experience in caring for organ recipients**	No	115	9.2	1.3	-1.66	0.10	
	Have	77	9.4	1.1			
**Is there a note to sign the organ donation**	No	95	9.2	1.4	2.41	0.09	
	Signed but no note	37	9.1	1.1			
	Has a signed card and is already under construction	60	9.6	1.0			
**Have attended organ donation related courses**	Participated	171	9.4	1.1	2.89	0.01	
	Never participated	21	8.3	1.6			

### Relationship between participant demographics and success in soliciting organ donations

Table 3 The relationship between nine variables—educational level, religious beliefs, work department, job title, type of hospital of employment, experience in caring for organ donors, experience in caring for organ recipients, organ donor registration status, and attendance at organ donation courses—comparing between participants who had and had not engaged in organ donation solicitation (*p*<0.05) The complete form is as S3 Table in the attached information. Table 4: Multiple regression analysis results indicated that the key factors for the successful solicitation of organ donations included “having experience in caring for organ donors,” “an organ donation coordinator job title,” and “having take organ donation courses.” Participants who were organ donation coordinators with experience in caring for organ donors and who attended organ donation courses had performed well in soliciting organ donations. Table 5 suggests that among organ donation course participants, the highest proportion attended courses on soliciting organ donations, followed by family grief counseling courses, organ transplant courses, and brain death determination courses.

**Table 3:** Relationship between basic attributes.

		None (N = 79) number (percent)	Yes (N = 113) number (percent)	Chi-square value	p-value
**Sex**	Male	8(10.1)	8(7.1)	0.565	0.452
	Female	71(89.9)	105(92.9)		
**Age**	<30 (i.e. 20–29)	10(12.7)	19(16.8)	2.142	0.343
	30–39	55(69.6)	67(59.3)		
	> = 40	14(17.7)	27(23.9)		
**Education level**	Specialist	11(13.9)	5(4.4)	8.683	0.013
	the University	55(69.6)	74(65.5)		
	Institute with above	13(16.5)	34(30.1)		
**Religion**	no	36(45.6)	35(31.0)	4.251	0.039
	Have	43(54.4)	78(69.0)		
**Work department**	Emergency	6(7.6)	1(0.9)	34.677	<0.001
	Surgery	21(26.6)	41(36.3)		
	Internal medicine	25(31.6)	17(15.0)		
	Obstetrics and Gynecology	4(100)	0(0)		
	Pediatrics	4(100)	0(0)		
	Intensive care unit	15(19.0)	28(24.8)		
	Other: Social Worker	4(5.1)	26(23.0)		
**Job title**	Medical staff (Physician, Nurse)	66(83.5)	33(29.2)	59.972	<0.001
	Organ Donation Coordinator	13(16.5)	80(70.8)		
**Type of hospital**	Medical center	35(44.3)	68(60.2)	21.435	<0.001
	Regional hospital	24(30.4)	41(36.3)		
	District hospital	13(16.5)	4(3.5)		
	Primary care	7(100)	0(0)		
**Take care of organs Donor experience**	no	60(75.9)	39(34.5)	31.963	<0.001
	Have	19(24.1)	74(65.5)		
**Experience in caring for organ recipients**	no	55(69.6)	60(53.1)	5.284	0.022
	Have	24(30.4)	53(46.9)		
**Is there a note Sign organ donation**	no	44(55.7)	51(45.1)	15.574	<0.001
	Signed card but no note	22(27.8)	15(13.3)		
	Signed card and note of insurance card under construction	13(16.5)	47(41.6)		
**Have attended organ donation related courses**	participated	62(78.5)	109(96.5)	15.429	<0.001
	Never participated	17(21.5)	4(3.5)		

**Table 4:** Regression results for basic attributes with regard to soliciting organ donations.

	Estimate of regression coefficient β	Standard error (S.E.)	Distinctiveness	Odds ratio
**Constant**	−7.304	1.392	0.000	0.001
**Education level**				
**Specialist**	0.392	0.798	0.594	1.433
**University and Technical College**	0.410	0.831	0.621	1.507
**Institute with above**	0.792	0.888	0.373	2.207
**Experience in caring for donors (yes)**	2.096	0.546	<0.001	8.135
**Experience in caring for recipients (yes)**	−0.582	0.560	0.299	0.559
**Whether to sign an organ donation**				
**No card**	**−0.477**	**0.510**	**0.349**	**0.607**
**With card/but not noted**	−0.478	0.512	0.351	0.620
**With Card/Annotated Health Insurance Card**	0.154	0.509	0.762	1.167
**Title (Organ Donation Coordinator)**	2.390	0.427	<0.001	10.911
**Belief (yes)**	0.468	0.406	0.249	1.596
**Attend related courses (yes)**	1.497	0.747	0.045	4.469

**Table 5:** Relationship between basic attributes and participation in organ counseling courses.

Course	Participation	None (N = 79) number (percent)	Yes (N = 113) number (percent)	Chi-square value	p-value
**1 Organ counseling series**	No	30(38.0)	13(11.5)	18.744	<0.001
	Have	49(62.0)	100(88.5)		
**2 Cerebral death judgment course**	No	47(59.5)	37(32.7)	13.52	<0.001
	Have	32(40.5)	76(67.3)		
**3 Organ transplant courses**	No	42(53.2)	36(31.9)	8.75	0.003
	Have	37(46.8)	77(68.1)		
**4 Family grief coaching courses**	No	44(55.7)	26(23.0)	21.444	<0.001
	Have	35(44.3)	87(77.0)		

## Discussion and study limitations

### Discussion

1. The study participants were primarily medical staff and organ donation coordinators. The results revealed that those in surgical disciplines, women, organ donation coordinators, and medical center staff formed the majority of participants, Their age ranged from 30 to39 years old. They were mostly college or technical school graduates. Moreover, the average score for healthcare workers’ and organ donation coordinators’ attitudes toward organ donations was 78.1 ± 8.8, which signified that the participants generally had positive attitudes toward organ donation. In-depth discussions revealed that a majority of participants identified positively with organ donation but continued to worry about it, Clinical nursing staff may be reluctant to suggest organ donation to patients’ families because of their own unclear knowledge of organ donation, unwillingness to become involved with families’ grief and suffering, unacceptance of organ donations helping other patients and their families, worries that they are not empowered to solicit organ donations, fear of being blamed or refused, or potential conflicts from believing that organ donation means giving up medical treatment [13,14].
2. The participants scored 9.27 (SD = 1.21) in average on their knowledge of organ donation, which was higher than the average score from the public [13,14]. This result revealed the relationship between participants’ education and work experience as well as the participants’ continued learning with regard to organ donation. Question “I know the procedure for initiating the organ donation process,” had the fewest number of yes responses, which suggested that participants were unclear on how to initiate the organ donation process and continued to exhibit unfamiliarity with practical operations. Second, clinical first-line caregivers, who are key stakeholders for discovering potential organ donors, were the second highest group to answer incorrectly in question “I know the standards for confirming organ donors.” This point should be reinforced in organ donation education and advocacy.
3. Successful solicitations of organ donations revealed that among the 79 participants (41.1%). This is similar to the current medical environment. Because of the inability of medical care regulations to effectively protect practitioners and allay fears of medical disputes, many practitioners adopt conservative attitudes. Beginning the organ solicitation process from a medical perspective is suggested: Once a patient is suspected to be brain dead, the hospital must report the potential organ donation to the Organ Donation Association or the Ministry of Health and Welfare. Then, an organ donation coordinator should be requested to provide medical and administrative assistance in the hospital where the alleged brain-dead patient is hospitalized. Departments that declare organ donations should be rewarded at the end of the year to motivate medical staff and organ donation coordinators to discover potential donors.
4. Regarding participants’ attitude toward organ donation, 50.6% of participants had organ donor cards. This percentage was higher than that of nursing staff noted in the literature in Taiwan, which suggested high acceptance of the promotion of organ donation in recent years. Ke et al. [15] argued that because Taiwanese people are more restrained when expressing emotions and organ donation is mostly jointly decided by family members, they are more willing to choose donations if they know that the patient intends to donate their organs. That is why, Taiwan is currently promoting organ donor cards and marking people’s willingness to donate on health insurance cards to signify their support for organ donation.
5. Successes in soliciting organ donations had a positive correlation among nine variables—level of education, religious beliefs, work department, job title, type of hospital of employment, experience in caring for organ donors, experience in caring for organ recipients, organ donor registration status, and take at organ donation courses. These correlations were related to participants continuing to learn about organ donations through education and work experiences. Courses on soliciting organ donations had the highest significance among organ donation course participants, followed by family grief counseling courses, organ transplant courses, and brain death determination courses. These results indicated that the courses were beneficial to soliciting organ donations. Including courses of soliciting organ donations into compulsory course credits for medical staff and organ donation coordinators may improve organ donation solicitation.
6. Participants’ demographic traits and other factors influenced organ donation solicitation. To understand the factors influencing participants on organ donation results, multiple regression analysis was conducted on the nine variables of level of education, religious beliefs, work department, job title, type of hospital of employment, experience in caring for organ donors, experience in caring for organ recipients, organ donor registration status, and attendance at organ donation courses. The results revealed that key factors in the successful solicitation of organ donations included “having experience in caring for organ donors,” “an organ donation coordinator job title,” and “having attended organ donation courses.” These results revealed that organ donation coordinators with experience in caring for organ donors who attended organ donation courses had high performance results in soliciting organ donations. This was possibly because organ donation coordinators must undergo training and pass an exam to be certified and thus have more experience in soliciting donations. Medical staff should be encouraged to undergo such training.

### Study limitations

This study was conducted using a structured questionnaire to survey participants, who may have had reservations about or grossly misinterpreted the questionnaire contents; this may have led to measurement errors and due to differences in hospital scale and environment, only superficial study results.

## Conclusion and suggestions

### Suggestions

Hospitals soliciting organ donations implement operations on hospice care consultations for end-of-life patients to advocate for such patients to have the right to express intentions of donating their organs before actively inquiring for patients’ intentions on organ donation to spur major hospitals throughout Taiwan to actively join in organ donation solicitation. Medical staff are crucial to the organ donation process because they are on the frontlines in discovering potential organ donors. The attitude of medical staff toward organ donation affects the push for organ donations, and care throughout the entire organ donation process is a vital link [16]. How nursing staff can be encouraged to engage in soliciting organ donations are questions deserving attention. Therefore, the researchers of this study recommend the following: (1) Substantiate these findings regarding administrative practices as a reference for training courses on clinical planning and organ donation to encourage intensive care nurses to proactively solicit organ donations and increase the organ donation rate. (2) Include these training courses on organ donation in certifying exams for intensive care training and as the basis for counselors engaging in soliciting organ donations. This would reinforce intensive care nurses’ knowledge of and positive attitudes toward organ donation and increase willingness to solicit organ donations, which can ensure that patients on organ wait lists can benefit from the generosity of donors and their families. Furthermore, (3) as caregivers, emergency and critical care nurses are generally passive in soliciting organ donations; however, they also play the role of educators and organ donation solicitors. The results of nurses engaged in soliciting organ donations can be provided for future researchers studying the attitudes and behavior of intensive care nurses toward soliciting organ donations. These studies may improve the nursing and communication skills of nurses in emergency and critical units, which may allow them to allocate time when patients’ vital signs are stable for family members to consider and decide on organ donation [17].

### Conclusion

In Taiwan, because the organ transplant technology has continually evolved, the survival rate within one year after each transplant surgery has currently exceeded 80%, and transplantation has become a major treatment option for patients with organ failure. However, because of the conservative mindsets in the ethnic Chinese society, the lack of sources of transplanted organs is a major problem that must be overcome. Because changing people’s concepts on organ donation is a slow process, education plays a crucial role. Following the rapid evolution of technology, young people have come into contact with electronic products, and they have spent more time in virtual worlds than with their families and friends. In the society dominated by utilitarianism, which emphasizes rapid response to things without emotions, showing insufficient care for others has been a widespread phenomenon. Medical and nursing students are faced with the most immediate problems in human life in their future career. Therefore, life education plays a critical role in teaching ultimate concern and empathy in medical students, nursing students, and students of other types. Topics related to organ donation have been integrated in the courses at schools of all levels to instruct students with the appropriate concepts, knowledge, and life education meanings on organ donation, thereby promoting the general public’s approval for organ donation. Organ donation enables patients with organ failure to extend their life span and improves the quality of their life. Such is a new understanding of the eternity. Moreover, practical clinical knowledge and skills must continue to systematically promote the idea of organ donation. Support groups related to life education can be formed, and people are encouraged to share their life stories to continue reinforcing and sustaining the benefits of organ donation [18–21].

## Supporting information

S1 Table Factors related to participants’ basic attributes and attitudes toward organ donation. (DOCX)

S2 Table Factors related to participants’ basic attributes and knowledge of organ donation behavior (*N* = 192). (DOCX)

S3 Table Relationship between basic attributes. (DOCX)
