# Supplementary material for: Soliciting organ donations by medical personnel and organ donation coordinators: A factor analysis
Source: PLoS One. 2021 Apr 23;16(4):e0250249. doi: 10.1371/journal.pone.0250249 (PMC8064528; doi:10.1371/journal.pone.0250249)
Supplement: S1 Table — (DOCX) [file pone.0250249.s001.docx]

**Supplementary file Table 1. Factors related to participants’ basic attributes and attitudes toward organ donation.**

| **Basic attributes** | | **Number** | **Average** | **Standard deviation** | **T value/F value** | **P value** | **Post hoc comparison test** |
| --- | --- | --- | --- | --- | --- | --- | --- |
| Sex | Male | 16 | 80.2 | 8.3 | 0.971 | 0.333 |  |
|  | Female | 176 | 77.9 | 8.9 |  |  |  |
| Age | <30 (i.e. 20-29) | 29 | 76.0 | 9.5 | 0.977 | 0.378 |  |
|  | 30-39 | 122 | 78.6 | 8.1 |  |  |  |
|  | >=40 | 41 | 78.2 | 10.5 |  |  |  |
| Education level | Specialist | 16 | 76.8 | 10.0 | 0.971 | 0.381 |  |
|  | the University | 129 | 77.8 | 8.6 |  |  |  |
|  | The institute contains the above | 47 | 79.6 | 9.2 |  |  |  |
| Religious belief | no | 71 | 77.8 | 9.7 | -0.442 | 0.659 |  |
|  | Have | 121 | 78.4 | 8.4 |  |  |  |
| Marital status  Divorce or | unmarried | 92 | 77.1 | 8.4 | 1.391 | 0.251 |  |
|  | Married | 98 | 79.0 | 9.2 |  |  |  |
|  | separation | 2 | 83.5 | 6.4 |  |  |  |
| Department of work | ①Emergency | 7 | 76.1 | 9.0 | 0.41 | 0.872 |  |
|  | ②Surgery | 62 | 78.7 | 10.0 |  |  |  |
|  | ③Internal medicine | 42 | 78.5 | 9.3 |  |  |  |
|  | ④Obstetrics and gynecology | 4 | 73.3 | 5.7 |  |  |  |
|  | ⑤Pediatrics | 4 | 76.0 | 7.2 |  |  |  |
|  | ⑥Intensive care unit | 43 | 78.1 | 7.5 |  |  |  |
|  | ⑦Other: social worker | 30 | 77.5 | 8.3 |  |  |  |
| Working years | <1 | 3 | 84.7 | 6.7 | 1.155 | 0.328 |  |
|  | 1-3 | 15 | 76.3 | 9.45 |  |  |  |
|  | 3-5 | 17 | 75.9 | 8.8 |  |  |  |
|  | >5 | 157 | 78.4 | 8.8 |  |  |  |
| Job title | Medical staff (physician, nurse) | 99 | 78.0 | 8.4 | -0.177 | 0.859 |  |
|  | Organ donation coordinator | 93 | 78.3 | 9.3 |  |  |  |
| Type of hospital | ①Medical center | 103 | 77.7 | 9.0 | 1.444 | 0.231 |  |
|  | ②Regional hospital | 65 | 79.5 | 8.4 |  |  |  |
|  | ③District hospital | 17 | 77.7 | 9.8 |  |  |  |
|  | ④Primary care | 7 | 72.7 | 7.1 |  |  |  |
| Take care of organs donor experience | No | 115 | 77.5 | 8.7 | -1.109 | 0.269 |  |
|  | Have | 77 | 79.0 | 9.1 |  |  |  |
| Experience in caring for organ recipients | No | 99 | 77.4 | 8.3 | -1.166 | 0.245 |  |
|  | Have | 93 | 78.9 | 9.4 |  |  |  |
| whether they are registered organ donors | ①no | 95 | 74.9 | 8.9 | 15.353 | <0.001 | ①v.s②(p = 0.004) |
|  | ②Signed card but no note | 37 | 80.0 | 7.5 |  |  | ①v.s.③(p<0.001) |
|  | ③Have a card and have a note on the health card | 60 | 82.1 | 7.7 |  |  |  |
| whether they attended organ donation courses | participated | 171 | 78.7 | 8.7 | 2.675 | 0.008 |  |
|  | Never participated | 21 | 73.3 | 8.5 |  |  |  |
