# Supplementary material for: Soliciting organ donations by medical personnel and organ donation coordinators: A factor analysis
Source: PLoS One. 2021 Apr 23;16(4):e0250249. doi: 10.1371/journal.pone.0250249 (PMC8064528; doi:10.1371/journal.pone.0250249)
Supplement: S2 Table — (DOCX) [file pone.0250249.s002.docx]

**Supplementary file Table 2. Factors related to participants’ basic attributes and knowledge of organ donation behavior (*N* = 192).**

| **Basic attributes** | **Number** | **Average** | **Standard deviation** | | **T value/F value** | **P value** | **Post hoc comparison test** |
| --- | --- | --- | --- | --- | --- | --- | --- |
| Sex | Male | 16 | 9.3 | 0.9 | -0.31 | 0.94 |  |
|  | Female | 176 | 9.3 | 1.2 |  |  |  |
| Age | <30 (i.e. 20-29) | 29 | 9.3 | 1.3 | 0.76 | 0.47 |  |
|  | 30-39 | 122 | 9.2 | 1.3 |  |  |  |
|  | >=40 | 41 | 9.5 | 0.8 |  |  |  |
| Education level | Specialist | 16 | 8.6 | 1.6 | 2.82 | 0.06 |  |
|  | the University | 129 | 9.4 | 1.1 |  |  |  |
|  | The institute contains the above | 47 | 9.2 | 1.2 |  |  |  |
| Religious belief | no | 71 | 9.2 | 1.3 | -0.27 | 0.79 |  |
|  | Have | 121 | 9.3 | 1.2 |  |  |  |
| Marital status  Divorce or | unmarried | 92 | 9.2 | 1.3 | 0.64 | 0.53 |  |
|  | Married | 98 | 9.3 | 1.1 |  |  |  |
|  | separation | 2 | 10.0 | 0.0 |  |  |  |
| Department of work | ①Emergency | 7 | 8.6 | 1.6 | 7.97 | <0.001 | ④v.s. ①(p = 0.019) |
|  | ②Surgery | 62 | 9.5 | 1.1 |  |  | ④v.s. ②(p<0.001) |
|  | ③Internal medicine | 42 | 9.0 | 1.0 |  |  | ④v.s. ③(p<0.001) |
|  | ④Obstetrics and gynecology | 4 | 6.3 | 0.5 |  |  |  |
|  | ⑤Pediatrics | 4 | 8.3 | 1.9 |  |  | ④v.s. ⑥(p<0.001) |
|  | ⑥Intensive care unit | 43 | 9.4 | 1.3 |  |  | ④v.s. ⑦(p<0.001) |
|  | ⑦Other: social worker | 30 | 9.7 | 0.7 |  |  |  |
| Working years | <1 | 3 | 8.7 | 1.2 | 0.77 | 0.51 |  |
|  | 1-3 | 15 | 9.2 | 1.6 |  |  |  |
|  | 3-5 | 17 | 9.6 | 1.1 |  |  |  |
|  | >5 | 157 | 9.3 | 1.2 |  |  |  |
| Job title | Medical staff (physician, nurse) | 99 | 8.7 | 1.4 | −4.82 | <0.001 |  |
|  | Organ donation coordinator | 93 | 9.7 | 0.8 |  |  |  |
| Type of hospital | ①Medical center | 103 | 9.4 | 1.0 | 7.81 | <0.001 | ④v.s. ①(p<0.001) |
|  | ②Regional hospital | 65 | 9.3 | 1.3 |  |  | ④v.s. ②(p<0.001) |
|  | ③District hospital | 17 | 9.0 | 1.3 |  |  | ④v.s. ③(p = 0.007) |
|  | ④Primary care | 7 | 7.3 | 1.6 |  |  |  |
| Take care of organs donor experience | No | 99 | 8.9 | 1.3 | −4.23 | <0.001 |  |
|  | Have | 93 | 9.6 | 1.0 |  |  |  |
| Experience in caring for organ recipients | no | 115 | 9.2 | 1.3 | -1.66 | 0.10 |  |
|  | Have | 77 | 9.4 | 1.1 |  |  |  |
| Is there a note to sign the organ donation | no | 95 | 9.2 | 1.4 | 2.41 | 0.09 |  |
|  | Signed but no note | 37 | 9.1 | 1.1 |  |  |  |
|  | Has a signed card and is already under construction | 60 | 9.6 | 1.0 |  |  |  |
| Have attended organ donation related courses | Participated | 171 | 9.4 | 1.1 | 2.89 | 0.01 |  |
|  | Never participated | 21 | 8.3 | 1.6 |  |  |  |
