# Supplementary material for: Soliciting organ donations by medical personnel and organ donation coordinators: A factor analysis
Source: PLoS One. 2021 Apr 23;16(4):e0250249. doi: 10.1371/journal.pone.0250249 (PMC8064528; doi:10.1371/journal.pone.0250249)
Supplement: S3 Table — (DOCX) [file pone.0250249.s003.docx]

**Supplementary file Table 3. Relationship between basic attributes.**

| **Basic attributes** |  | **None (N = 79) Number (percent)** | **Yes (N = 113) Number (percent)** | **Chi-square value** | **p-value** |
| --- | --- | --- | --- | --- | --- |
| Sex | Male | 8(10.1) | 8(7.1) | 0.565 | 0.452 |
|  | Female | 71(89.9) | 105(92.9) |  |  |
| Age | <30 (i.e. 20-29) | 10(12.7) | 19(16.8) | 2.142 | 0.343 |
|  | 30-39 | 55(69.6) | 67(59.3) |  |  |
|  | >=40 | 14(17.7) | 27(23.9) |  |  |
| Education level | Specialist | 11(13.9) | 5(4.4) | 8.683 | 0.013 |
|  | The University | 55(69.6) | 74(65.5) |  |  |
|  | Institute with above | 13(16.5) | 34(30.1) |  |  |
| Religion | no | 36(45.6) | 35(31.0) | 4.251 | 0.039 |
|  | Have | 43(54.4) | 78(69.0) |  |  |
| marital status | unmarried | 39(54.4) | 53(46.9) | 3.147 | 0.207 |
|  | married  Divorce or | 38(48.1) | 60(53.1) |  |  |
|  | separation | 2(2.5) | 0(0) |  |  |
| Work department | Emergency | 6(7.6) | 1(0.9) | 34.677 | <0.001 |
|  | Surgery | 21(26.6) | 41(36.3) |  |  |
|  | Internal medicine | 25(31.6) | 17(15.0) |  |  |
|  | Obstetrics and Gynecology | 4(100) | 0(0) |  |  |
|  | Pediatrics | 4(100) | 0(0) |  |  |
|  | Intensive care unit | 15(19.0) | 28(24.8) |  |  |
|  | Other: social worker | 4(5.1) | 26(23.0) |  |  |
| Working years | <1 | 1(1.3) | 2(1.8) | 0.376 | 0.945 |
|  | 1-3 | 6(7.6) | 9(8.0) |  |  |
|  | 3-5 | 6(7.6) | 11(9.7) |  |  |
|  | >5 | 66(83.5) | 91(80.5) |  |  |
| Job title | Medical staff (physician, nurse) | 66(83.5) | 33(29.2) | 59.972 | <0.001 |
|  | Organ donation coordinator | 13(16.5) | 80(70.8) |  |  |
| Type of hospital | Medical center | 35(44.3) | 68(60.2) | 21.435 | <0.001 |
|  | Regional hospital | 24(30.4) | 41(36.3) |  |  |
|  | District hospital | 13(16.5) | 4(3.5) |  |  |
|  | Primary care | 7(100) | 0(0) |  |  |
| Take care of organs donor experience | no | 60(75.9) | 39(34.5) | 31.963 | <0.001 |
|  | Have | 19(24.1) | 74(65.5) |  |  |
| Experience in caring for organ recipients | no | 55(69.6) | 60(53.1) | 5.284 | 0.022 |
|  | Have | 24(30.4) | 53(46.9) |  |  |
| Is there a note sign organ donation | no | 44(55.7) | 51(45.1) | 15.574 | <0.001 |
|  | Signed card but no note | 22(27.8) | 15(13.3) |  |  |
|  | Signed card and note of insurance card under construction | 13(16.5) | 47(41.6) |  |  |
| Have attended organ donation related courses | participated | 62(78.5) | 109(96.5) | 15.429 | <0.001 |
|  | Never participated | 17(21.5) | 4(3.5) |  |  |
